# Therapeutic Efficacy of a *Trichinella Spiralis* Paramyosin-Derived Peptide Modified With a Membrane-Targeting Signal in Mice With Antigen-Induced Arthritis

**DOI:** 10.3389/fmicb.2020.608380

**Published:** 2020-12-23

**Authors:** Yi Chen, Shuai Shao, Jingjing Huang, Yuan Gu, Yuli Cheng, Xinping Zhu

**Affiliations:** Department of Medical Microbiology and Parasitology, School of Basic Medical Sciences, Capital Medical University, Beijing, China

**Keywords:** *Trichinella spiralis*, paramyosin, complement C9, antigen-induced arthritis, modified peptide, therapeutic efficacy

## Abstract

Helminth-derived molecules have the ability to modulate the host immune system. Our previous study identified a tetradecapeptide derived from *Trichinella spiralis* paramyosin (*Ts*-pmy) that could bind to human complement component C9 to inhibit its polymerization, making the peptide a candidate therapeutic agent for complement-related immune disorders. Here, the peptide underwent an N-terminal modification with a membrane-targeting signal (a unique myristoylated peptide) to improve its therapeutic efficacy. We found that the modified peptide had a binding affinity to human C9 that was similar to that of the original peptide, as confirmed by microscale thermophoresis assays. The binding of the modified peptide to human C9 resulted in the inhibition of C9-related complement activation, as reflected by the decreased Zn^2+^-induced C9 polymerization and the decreased C9-dependent lysis of rabbit erythrocytes. In addition, the original and modified peptides could both bind to recombinant mouse C9 and inhibit the C9-dependent lysis of rabbit erythrocytes in normal mouse serum (NMS), which meant that the peptides could cross the species barrier to inhibit complement activity in mice. Further *in vitro* and *in vivo* analyses confirmed that the peptide modification increased the retention time of the peptide. Furthermore, intraarticular injection of the modified peptide markedly ameliorated knee swelling and joint damage in mice with antigen-induced arthritis (AIA), as assessed histologically. These results suggested that the *Ts*-pmy-derived peptide modified with a membrane-targeting signal was a reasonable candidate therapeutic agent for membrane attack complex (MAC)-related diseases [such as rheumatoid arthritis (RA)] and the study presented a new modification method to improve the potential therapeutic effects of the peptide.

## Introduction

During the long coevolution of hosts and parasites, parasites developed a variety of proteins to allow them to escape from host immune attack (Song et al., 2018; Xu et al., 2018; Conigliaro et al., 2019; Shao et al., 2019). These parasite-derived proteins can regulate the host immune response by interacting with key molecules of the immune system (Zhang et al., 2011; Zhao et al., 2017; Shao et al., 2019). Because of their strong immunomodulatory functions, some helminth-derived proteins are sufficiently potent to treat a variety of allergies, autoimmune diseases, and other immune disorders (Wang et al., 2017; Wu et al., 2017). The complement system, consisting of more than 25 proteins, plays vital roles in defending against infections and is an important component of both innate and adaptive immunity (Dijkstra et al., 2019). Due to the complex impacts of the complement system on the immune system, it is a double-edged sword. A certain level of activation of the complement system facilitates pathogen elimination by opsonizing pathogens, promoting chemotaxis of immune cells and directly damaging pathogen surfaces (Conigliaro et al., 2019). However, overactivation of the complement system contributes to the pathogenesis of many human diseases, including rheumatoid arthritis (RA) (Bemis et al., 2019) and atypical hemolytic uremic syndrome (Rondeau et al., 2020). In these diseases, autoantibodies are deposited in normal tissues in the body and they activate the complement system, resulting in local inflammation and cell injury. It has been reported that helminth-derived proteins interact with the key components of the complement system to inhibit complement activation as the initial step of the evasion of host immune clearance (Shao et al., 2019). These proteins also have promising therapeutic potential for treating inflammatory diseases related to disorders of complement activation (Wang et al., 2017; Wu et al., 2017).

Rheumatoid arthritis (RA) is a chronic inflammatory disease characterized by the infiltration of peripheral inflammatory cells and the proliferation of synovial lining cells in peripheral joints, which results in swollen and tender joints followed by cartilage and bone destruction (Aletaha, 2020). Although there are differing opinions about the etiology of RA, it is widely accepted that autoantibody-induced complement activation plays an important role (Trouw et al., 2017). Many studies support the association between complement activation and the pathogenesis of RA (Holers and Banda, 2018; Bemis et al., 2019). Complement activation can be triggered through the classical, lectin and alternative pathways and then participate in the control of the immune reaction by producing a series of intermediate products such as C3b, C3a, C4b, and membrane attack complex (MAC) (Shao et al., 2019). As the terminal component arising from complement activation, MAC, which is composed of C5b, C6, C7, C8, and several C9 molecules, has been shown to contribute to the pathogenesis of RA owing to its unique biological functions (Darrah and Andrade, 2018). Once the complement system is activated by autoantibodies within the joint cavity, C5b is produced by cleavage of C5 catalyzed by a C5 convertase (Bemis et al., 2019). C5b subsequently binds to the lipid outer surface of nearby living cells and finally assembles MAC, a transmembrane channel pore, on the cell surface (Shao et al., 2019). MAC increases cell proliferation and cytokine secretory capacity, which are associated with the pathogenesis of RA, making MAC a potential therapeutic target for easing the symptoms of RA (Morgan, 2016; Xie et al., 2020).

As MAC is a therapeutic target in RA, complement inhibitors have shown potential for preventing and treating arthritis in animal models (Goodfellow et al., 2000; Durigutto et al., 2013). Moreover, local or targeted inhibition might be an effective way to circumvent the adverse effects of systemic complement inhibition. For instance, CD59, a heavily glycosylated protein of 20 kDa that can specifically inhibit MAC formation, has been assessed as a treatment for RA and shown good effects in some animal experiments. More precisely, intraarticular injection of membrane-targeted CD59 or Fc fusion proteins containing CD59 in place of the Fab arms of the antibody markedly ameliorated the severity of antigen-induced arthritis (AIA) in mice, indicating the therapeutic efficacy of CD59 in RA (Harris et al., 2002; Williams et al., 2004).

Paramyosin, a dimeric fibrillar protein, not only forms the thick myofilaments of invertebrate muscle, but also occurs on the surface of helminths and serves as a potential modulator of the host immune system by interacting with human collagen, calgranulin and so on (Zhao et al., 2014). Our previous study revealed that *Trichinella spiralis* paramyosin (*Ts*-pmy), which is expressed on the outer membrane of *T. spiralis*, could bind to C9 and inhibit MAC assembly (Zhang et al., 2011). Furthermore, fragmental expression and synthesized peptide screening precisely identified that 14 amino acids (VSMGKSLSSKVYVM) made up the C9-binding site in the C-terminus of *Ts*-pmy (Zhao et al., 2014). Thus, this *Ts*-pmy-derived peptide was confirmed to be a MAC inhibitor-like CD59, and it might have therapeutic efficacy when it is injected into articular cavities. In this study, a membrane-targeting signal was linked to the C-terminus of the *Ts*-pmy-derived peptide. Thereafter, the biological function of the modified peptide and its therapeutic effects on AIA were evaluated *in vitro* and *in vivo*. It was confirmed that the *Ts*-pmy-derived peptide modified with the membrane-targeting signal can serve as a potential complement inhibitor in the treatment of AIA.

## Materials and Methods

### Animals

Ten-week-old male C57BL/6J wildtype mice were purchased from the Laboratory Animal Services Center of Capital Medical University (Beijing, China) and reared in a barrier facility at a suitable temperature(16–26°C) and humidity (40–70%). The animal experiments were approved by the Capital Medical University Animal Care and Use Committee (approval number: AEEI-2016-119) and were in accordance with the US National Institutes of Health (NIH) Guidelines for the Care and Use of Laboratory Animals.

### Sera

Normal human serum (NHS) was collected from six healthy volunteers, and the donation procedure was approved by the Institutional Review Board (IRB) of Capital Medical University (approval number: 2016SY01). After venous blood was collected, it was clotted in serum separator tubes for 4 h at room temperature and then centrifuged at 1,000 g for 30 min to obtain the serum. The serum was divided into aliquots and stored at −80°C. To obtain normal mouse serum (NMS), blood was collected in the oculomotor sinus of mice under ether anesthesia and the serum was separated and stored as described above.

### ATDC5 Cell Line

The mouse ATDC5 chondrogenic cell line is an important chondrocyte model that has been widely used in the study of chondrocyte proliferation and differentiation. This cell line was used to evaluate the membrane-binding ability of the modified peptide, as articular cartilage (which contains chondrocytes) is one of the main cellular components in the joint cavity. The cells were purchased from Guangzhou Jennio Biotech Co., Ltd. (Guangzhou, China) and maintained in Dulbecco's Modified Eagle Medium (DMEM, ThermoFisher, America) supplemented with 10% fetal bovine serum (FBS, Gibco, Australia) at 37°C in a humidified atmosphere with 5% CO_2_. The 5th−10th passages of the cells were used in the study.

### Peptide Synthesis

The original, modified, and control peptides were synthesized using solid-phase peptide synthesis by GL Biochem Co., Ltd (Shanghai, China). Briefly, 6-aminocaproic acid (ACP) was used as a linker between the membrane-targeting peptide (Myr-GSSKSPSKKKKKKPG) and the original peptide (VSMGKSLSSKVYVM), and the resultant peptide was designated the modified peptide (Myr-GSSKSPSKKKKKKPG-ACP-VSMGKSLSSKVYVM). The sequence of the original peptide was randomly rearranged to create the control peptide (GKMSVMSVYKSVLS). To assess the binding of these peptides to the cell membrane, they were labeled with biotin at the C-terminus. The resultant peptides were purified (up to 95%) by preparative reversed-phase (RP)-high-performance liquid chromatography (HPLC) and verified by mass spectrometry.

### Microscale Thermophoresis (MST) Assays

To assess the binding affinity of the peptides to human or mouse complement C9, a fluorescence-based biophysical technique known as MST (NanoTemper Technologies GmbH, Germany) was used. Briefly, human or mouse C9 was fluorescently labeled using a Monolith NT™ Protein Labeling Kit NT-647 (NanoTemper Technologies GmbH, Germany) based on the manufacturer's protocol. Next, the peptides were gradiently diluted in distilled water from 1.68 to 0 mM. The labeled human or mouse C9 (1.5 μM) was then incubated with various peptide concentrations for 10 min at room temperature before being loaded into K002 silica capillaries. Measurements were performed using a NanoTemper Monolith NT.115 instrument in distilled water containing 0.05% Tween 20. The data were analyzed using NanoTemper Analysis software (NanoTemper Technologies GmbH, Germany).

### C9 Polymerization Assays

Certain concentrations of C9 can polymerize when induced by certain concentrations of Zn^2+^. However, the polymerization can be suppressed when C9 binds to inhibitors. C9 polymerization assays were used to evaluate the inhibitory effect of the peptides on Zn^2+^-induced polymerization of human C9. Briefly, 2 μg C9 was preincubated with the peptides in 20 mM Tris buffer (pH 7.2) at 37°C for 40 min and then 50 μM Zn^2+^ was added to induce C9 polymerization at 37°C for 2 h. The samples were subjected to 2.5–15% polyacrylamide gel electrophoresis to assess the inhibitory effects of the peptides on human C9 polymerization; the gels were visualized by staining with Coomassie blue.

### Hemolytic Assays

When rabbit erythrocytes are placed in xenogeneic serum (such as NHS or NMS), the alternative pathway of the complement system is activated, causing lysis of the rabbit erythrocytes. To evaluate the effects of the peptides on the complement activation-mediated erythrocyte lysis (involving the alternative complement pathway), NHS or NMS (1:12.5) was incubated with various concentrations of peptides (1.31, 2.62, and 5.24 μmol) in Mg-EGTA solution (5 mM MgCl_2_, 10 mM EGTA) at 37°C for 40 min before adding the mixture to freshly washed erythrocytes (1 × 10^8^) in gelatin veronal-buffered saline (pH 7.4, containing 0.1% gelatin) for 30 min at 37°C. Lysis was stopped with cold Hanks' Balanced Salt Solution (HBSS^++^) containing 10 mM ethylenediaminetetraacetic acid (EDTA). The samples were then centrifuged at 1,000 g for 10 min to obtain the supernatant, and the amount of released hemoglobin in the supernatant was determined by measuring the absorbance at 412 nm. The hemolytic activity relative to the water-induced lysis among the same number of erythrocytes was calculated.

### Assessment of Peptide Retention on Cell Membrane

To assess the binding ability of the modified peptide to the cell membrane, the three peptides were labeled with biotin at the C-terminus. Cultured mouse ATDC5 cells were washed with phosphate-buffered saline (PBS) and incubated with various concentrations of the control, original or modified peptides (65, 130, and 260 nmol, respectively) for 1 h at room temperature. After washing twice with PBS to remove the unbound peptides, streptavidin-fluorescein isothiocyanate (FITC; eBioscience, California, USA) was added and incubated for 0.5 h at room temperature. The cells were washed and resuspended in PBS and then the binding of the peptides to the cell membrane was assessed by flow cytometry (BD Biosciences, California, USA). To further assess the retention of peptides on the surface of cells, the mouse ATDC5 cells were cultured in a 12-well culture plate which contains cell slides on the bottom. They were washed with PBS on the next day and then blocked with normal goat serum for 30 min at room temperature before incubation with various concentrations of peptides (260 nmol, 540 nmol, and 1.08 mmol). The binding of the peptides to the cell membrane was then assessed by staining with streptavidin-FITC (eBioscience). After being stained, the cells that had adhered to the cell slides were mounted using ProLong Gold Antifade reagent (ZSGB-BIO, Beijing, china) containing 4′,6-diamidino-2-phenylindole (DAPI, ThermoFisher, America) dye to stain the cell nuclei. They were then analyzed by confocal laser scanning microscopy (Leica, Germany).

### Assessment of Peptide Retention in Normal Mouse Joint Cavities

As previous studies reported, immunofluorescence assay was used to assess the retention time of the peptides in normal mouse joint cavities (Williams et al., 2004). The normal mice were divided into original and modified peptide groups and treated with the corresponding peptides [10 μg in 10 μl PBS by intraarticular (IA) injection respectively. At 12, 24, and 48 h after injection, a single mouse from each group was randomly chosen and sacrificed under anesthesia (1% sodium pentobarbital, 50 mg/kg)]. Knee joints were dissected, fixed in 4% paraformaldehyde, and decalcified with decalcification solution (0.5 M EDTA, pH 8.0) before embedding in paraffin. Midsagittal serial sections (7 μm in thickness) were cut and peptide retention was assessed using streptavidin-FITC (eBioscience) (1:100 in PBS) followed by fluorescent microscopy (Leica).

### Induction of AIA in Mice and Therapeutic Effect of Peptides on AIA

AIA was induced in male C57BL/6J wildtype mice (8–10 weeks of age) according to a previously described protocol (Ebbinghaus et al., 2017) with minor modifications. Briefly, the mice were intraperitoneally injected with 200 ng pertussis toxin (List Biological Laboratories, California, USA) prior to being subcutaneously immunized with 100 μg methylated bovine serum albumin (mBSA; Sigma-Aldrich, St. Louis, Missouri, USA) emulsified in Freund's complete adjuvant (Sigma-Aldrich). The immune response was boosted with identical subcutaneous mBSA immunizations at 14 and 21 days after the first immunization. At 28 days after the initial immunization, the right knee joint of each mouse was intraarticularly injected with 10 μl PBS as the control, while the left knee joint received either 10 μl PBS only (normal group), 25 μg mBSA (to induce AIA) in 10 μl PBS (AIA-only group), 40 μg modified peptide with 25 μg mBSA in 10 μl PBS (modified peptide+AIA group), or 40 μg original peptide with 25 μg mBSA in 10 μl PBS (original peptide+AIA group). Every day after AIA induction (and intraarticular peptide treatment), the difference in the joint diameter (swelling in mm) between the right and left knee joints in each mouse was assessed using a digital micrometer to evaluate the AIA severity. Each joint was measured three times and the mean diameter was calculated and recorded. For the statistical analysis, a minimum of five mice per group were used. All experiments were performed three times to ensure reproducibility of results.

### Histologic and Immunofluorescence Analyses

To assess the therapeutic effects of the peptides on AIA, the mice were euthanized on day 3 post-AIA induction. The knee joints were separated, fixed, decalcified, and embedded in paraffin as described above. After midsagittal serial sections (7 μm in thickness) were cut, the paraffin-embedded knee joint sections were stained with hematoxylin and eosin (HE). The degree of histopathologic tissue damage was quantified using the following previously described cumulative score for evaluating disease severity (Durigutto et al., 2013). Degree of synovial hyperplasia (1–2 lines of regularly shaped synoviocytes = 0; 3–5 lines of irregularly shaped synoviocytes = 1; >5 lines of hypertrophied cells = 2); leukocyte infiltration in synovial tissue (no infiltration = 0; scattered cells = 1; focal infiltration in up to 50% of the synovial tissue = 2); percent diffusion of tissue fibrosis (no alterations = 0; 25–50% loss of the fat tissue component of synovial membranes = 1; >50% loss of the fat tissue component of synovial membranes = 2). Additionally, according to the immunofluorescence protocol, tissue deposition of MAC was assessed by immunofluorescence assay with incubating rabbit anti-mouse C9 IgG (Sigma-Aldrich) at a 1:200 dilution overnight at 4°C followed by incubation with FITC-labeled goat anti-rabbit IgG (DAKO, Glostrup, Denmark) at a 1:200 dilution for an additional 60 min at room temperature (Lei et al., 2020). Subsequently, the sections were observed and photographed by microscopy (Leica, Germany).

### Statistical Analysis

GraphPad Prism version 6 software (GraphPad Software, San Diego, CA, USA) was used to statistically analyze the data. The results were presented as mean ± standard deviation. Statistical significance was determined by one-way analysis of variance (ANOVA) followed by Tukey's *post-hoc* tests; *P* < 0.05 was considered statistically significant.

## Results

### Binding of Modified Peptide to Human C9 Inhibits C9 Polymerization-Induced Bio-Function

**Figure 1:**
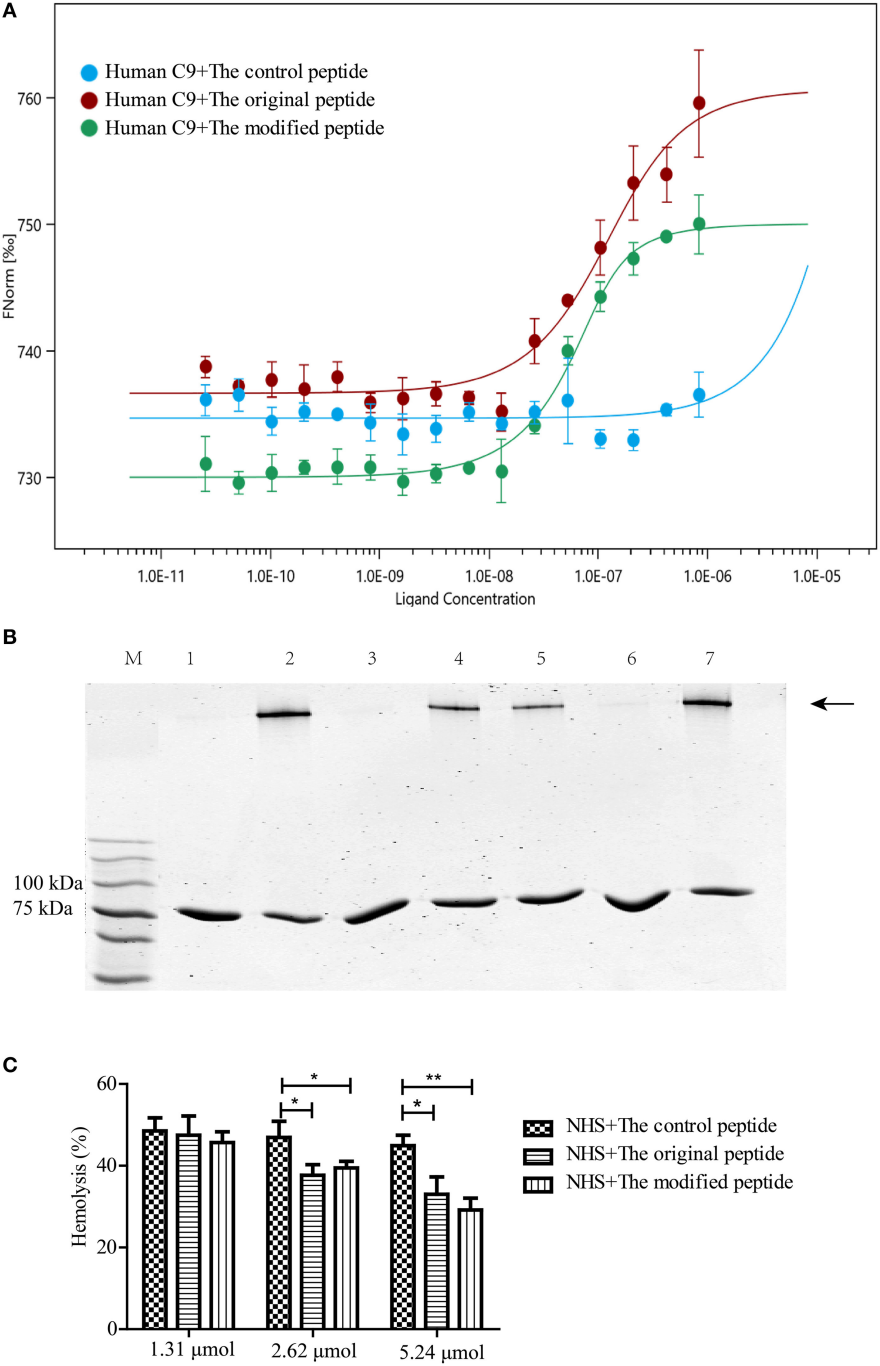
Modified peptide bound to human complement C9 and inhibited C9 polymerization-induced bio-function. **(A)** Microscale thermophoresis assay (NanoTemper) in which biomolecular interactions were quantitated by examining the motion of the molecules along a microscopic temperature gradient induced by an infrared laser. Changes in the molecular hydration shell, charge, or size are measured using a fluorescent probe (NT-647) bound covalently to the human C9. Binding of control, modified and original peptides to human C9 were studied. Data points indicate the differences in normalized fluorescence (%) generated by peptides binding to mouse C9, and curves indicate the calculated fits. There was a concentration-dependent binding between the human C9 and the original or modified peptide instead of the control peptide. Error bars represent the standard error of three independent measurements. **(B)** Zn^2+^-induced C9 polymerization (indicated by the arrowhead) was inhibited in a dose-dependent manner by the modified peptide [slightly by 2.62 μmol (lane 5) and completely by 5.24 μmol (lane 6)] compared to the control peptide [5.24 μmol (lane 7)] or the original peptide [5.24 μmol (lane 3)]. Lane M: standard protein markers; lane 1: blank control (C9 without Zn^2+^); lane 2: positive control (C9 with Zn^2+^); lane 3: 5.24 μmol original peptide; lanes 4–6, 1.31, 2.62, and 5.24 μmol modified peptide, respectively; lane 7, 5.24 μmol control peptide. **(C)** Normal human serum (NHS)-induced complement-mediated hemolysis was inhibited by the modified and original peptides (2.62 and 5.24 μmol) compared to the control peptide. All experiments were performed three times and data are presented as mean ± SD. *p < 0.05, **p < 0.01.

C9 polymerization is a prerequisite for MAC formation and its bio-function regarding eliminating pathogens. To confirm the binding between the peptides and human C9, MST assays were used to quantify the binding affinity. The MST results showed that human C9 could bind to both the original and modified peptides, leading to S-shaped curves being plotted (Figure 1A). However, there was no binding between human C9 and the control peptide, leading to a horizontal line being plotted (Figure 1A). To assess whether the modified peptide has the same ability as the original peptide to inhibit Zn^2+^-induced C9 polymerization, 2 μg C9 was preincubated with various concentrations of peptides prior to incubation with 50 μM Zn^2+^. As shown in Figure 1B, the modified peptide inhibited Zn^2+^-induced C9 polymerization in a dose-dependent manner. When the concentration of the modified peptide reached 5.24 μmol, the C9 polymerization was fully inhibited, as it was with 5.24 μmol original peptide. In contrast, 5.24 μmol control peptide did not inhibit C9 polymerization (Figure 1B). To further explore the inhibitory effects of the peptides on human C9 polymerization, NHS-induced complement-mediated erythrocyte lysis (involving the alternative pathway) was investigated. The erythrocyte lysis was significantly inhibited in a dose-dependent manner in the presence of the modified or original peptide, with the greatest inhibition occurring when the concentration of the modified or original peptide reached 5.24 μmol. However, there was no difference in the erythrocyte lysis among the three concentrations of control peptide (1.31, 2.62, and 5.24 μmol) (Figure 1C).

### Modified Peptide Binding to Mouse C9 Inhibits NMS-Induced Hemolysis of Rabbit Erythrocytes

**Figure 2:**
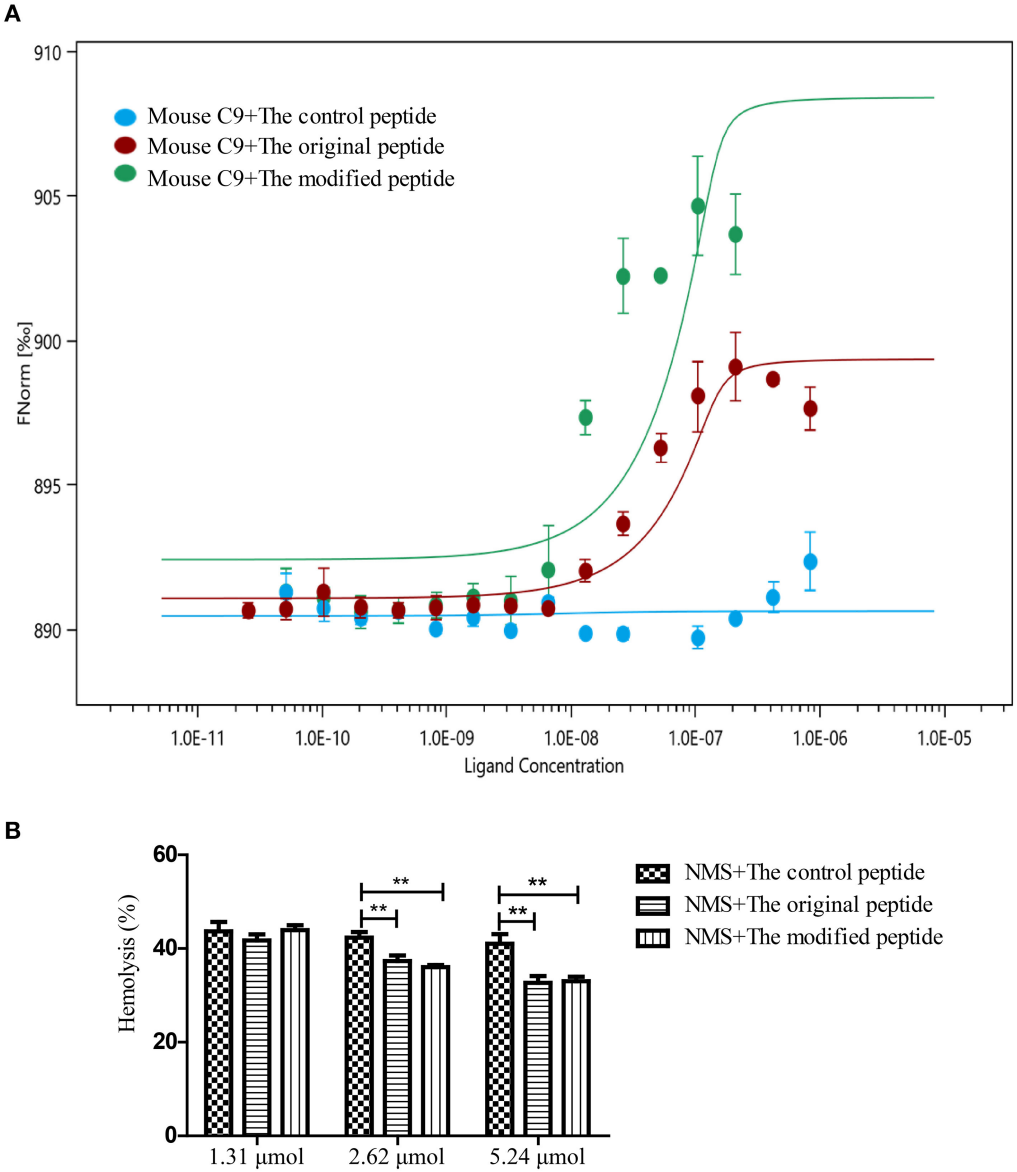
Modified peptide bound to mouse complement C9 and inhibited normal mouse serum (NMS)-induced hemolysis of rabbit erythrocytes. **(A)** Microscale thermophoresis assay (NanoTemper) in which biomolecular interactions are quantitated by examining the motion of the molecules along a microscopic temperature gradient induced by an infrared laser. Changes in the molecular hydration shell, charge, or size are measured using a fluorescent probe (NT-647) bound covalently to the mouse C9. Binding of control, modified and original peptides to mouse C9 were studied. There was a concentration-dependent binding between the mouse C9 and the original or modified peptide instead of the control peptide. Data points indicate the differences in normalized fluorescence (%) generated by peptides binding to mouse C9, and curves indicate the calculated fits. Error bars represent the standard error of three independent measurements. **(B)** NMS-induced complement-mediated hemolysis was inhibited by the modified and original peptides (2.62 and 5.24 μmol) compared to the control peptide. All experiments were performed three times and data are presented as mean ± SD. *p < 0.05, **p < 0.01.

To evaluate whether the modified peptide can cross the species barrier to inhibit murine MAC assembly in addition to human MAC assembly and to provide a preliminary experimental basis for the *in vivo* mouse experiments, MST assays were used again to assess the binding ability of the peptides to recombinant mouse C9. Recombinant mouse C9 was cloned, expressed, purified and refolded as soluble protein (data not shown). As shown in Figure 2, both the original and modified peptides could bind to the recombinant mouse C9, but the control peptide could not (Figure 2A). To evaluate whether this binding can inhibit NMS-induced erythrocyte lysis (involving the alternative complement pathway), hemolytic assays were performed. The results showed that erythrocyte lysis was inhibited in a dose-dependent manner in the presence of the modified or original peptide (Figure 2B). When the concentration of the modified or original peptide reached 5.24 μmol, the NMS-induced erythrocyte lysis was reduced, to a similar level to the NHS-induced erythrocyte lysis. However, the control peptide (as in the NHS-induced lysis assays) still showed no inhibitory effect (Figure 2B).

### Modified Peptide Can Nonspecifically Bind to Cell Membrane

**Figure 3:**
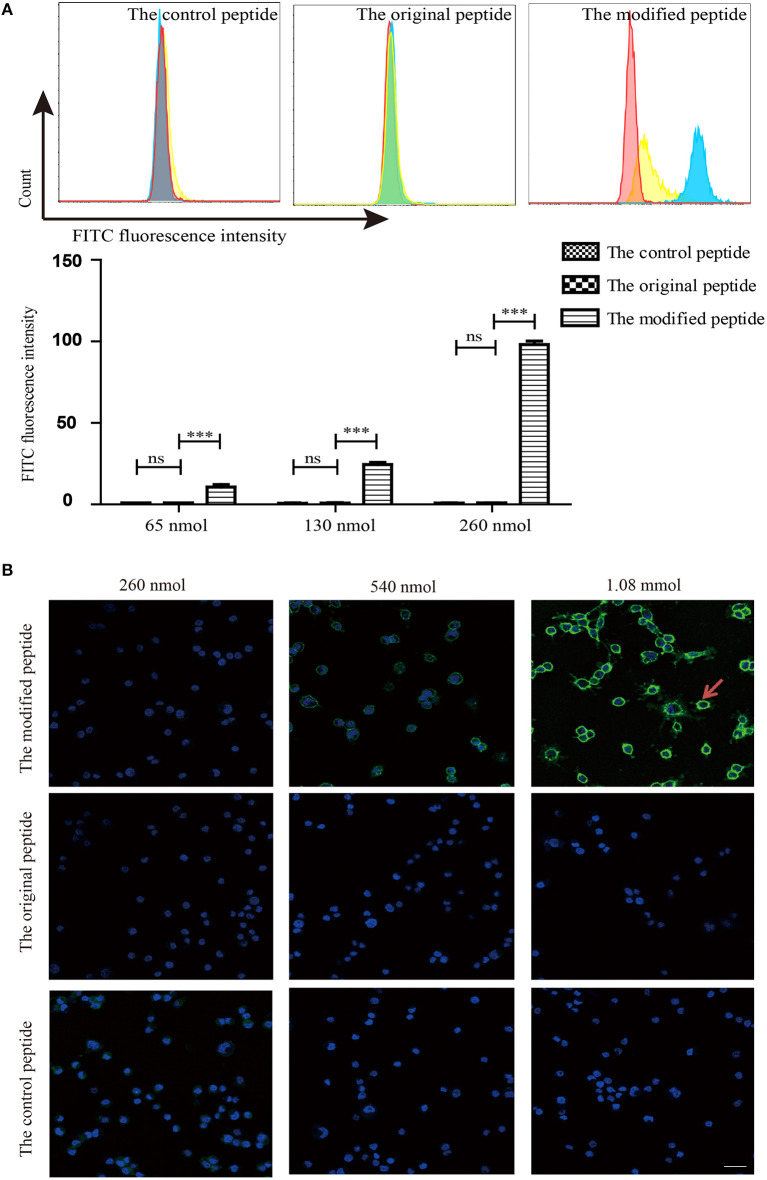
Modified peptide non-specifically bound to the cell surface. **(A)** Binding of peptides to the surface of mouse ATDC5 cells measured by flow cytometry. All experiments were performed three times and data are presented as mean ± SD. *n* = 5, ***p < 0.001, ns: not significant (*p* > 0.05). **(B)** Binding of various concentrations of biotin-labeled peptides on the surface of mouse ATDC5 cells assessed by confocal laser scanning microscopy. Arrowheads indicate the binding of the modified peptides on the surface of mouse ATDC5 cells.

To identify whether the membrane-targeting signal allows the modified peptide to bind to the cell membrane, mouse ATDC5 cells were cultured as the source of membrane and flow cytometry and confocal laser scanning microscopy were used to assess the binding of the peptides. The control, original, and modified peptides were labeled with biotin at the C-terminus so that they could be detected using streptavidin-FITC. As shown in Figure 3A, the modified peptide bound to the cell surface of mouse ATDC5 cells in a dose-dependent manner; the mouse ATDC5 cells that were bound to the modified peptide reached almost 100% when the concentration of modified peptide was 260 nmol. In contrast, the control and original peptides at the same concentration did not bind to the cell membrane. Similarly, the confocal laser scanning microscopy results in Figure 3B visibly revealed that the modified peptide could bind to the surface of mouse ATDC5 cells, further indicating the cell membrane-targeting properties of the modified peptide. The presence of the modified peptide on the cell surface suggested that it would be accessible to the host complement components if it was injected into a joint cavity as a possible complement inhibitor.

### Modified Peptide Has Prolonged Retention Time in Joint Cavities

**Figure 4:**
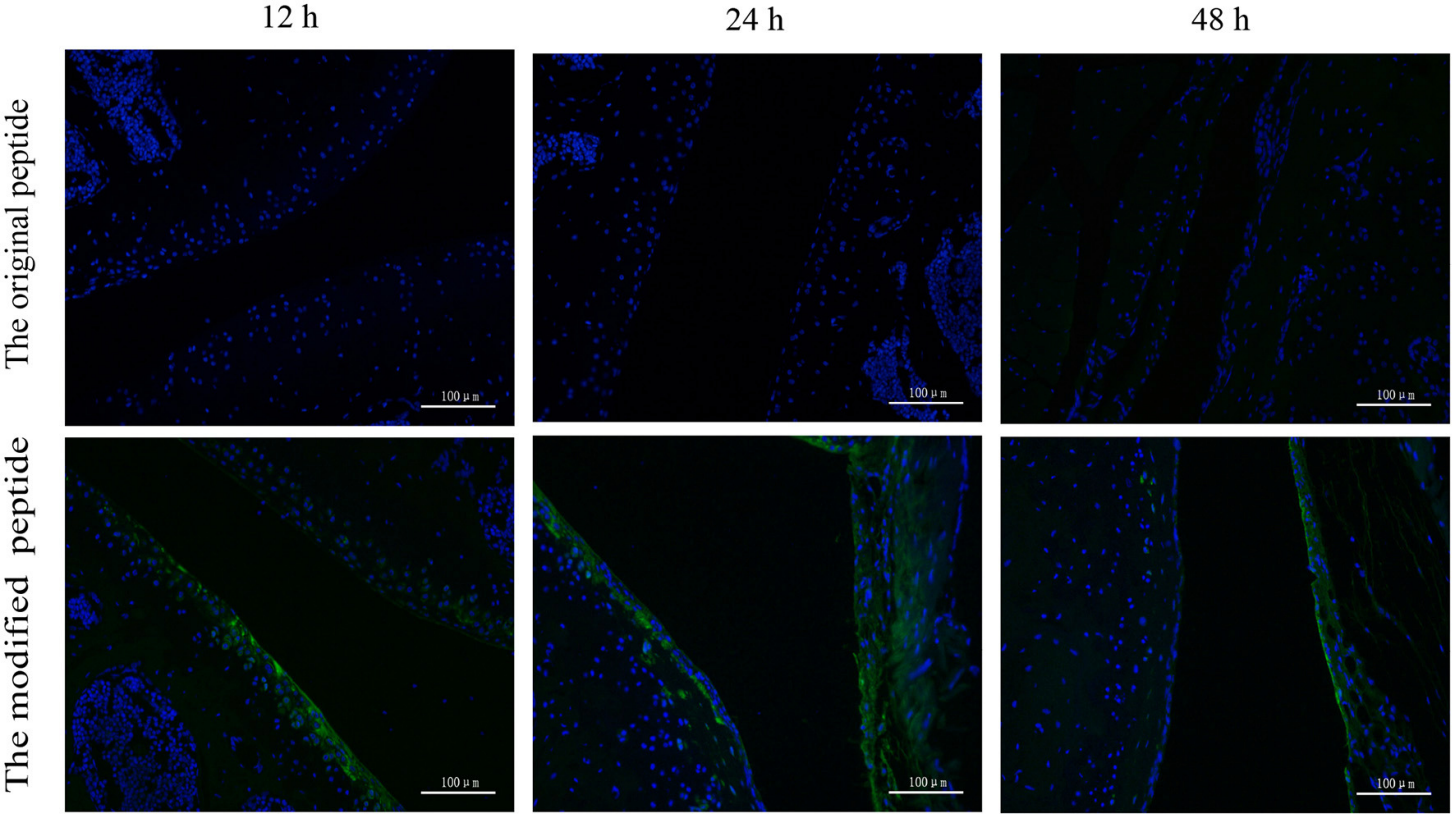
Retention time of the modified peptide in the joint cavities was prolonged. The biotin-labeled original or modified peptide was injected into the knee joint and then the knee joints were dissected at 12, 24, and 48 h and probed with streptavidin-FITC in immunofluorescence assays.

To assess the retention of peptides in normal mouse joints *in vivo*, the control, original, and modified peptides were injected into mice articular cavities. At 12, 24, and 48 h post injection, a mouse from each group was sacrificed to dissect the knee joint and then stain it with streptavidin-FITC. Compared to the joint cavity injected with original peptide, which showed no staining, the articular cavities injected with the modified peptide exhibited intense staining along the synovial lining even at 48 h after injection (Figure 4).

### Modified Peptide Alleviates AIA by Reducing MAC Deposition in the Joints of Mice

**Figure 5:**
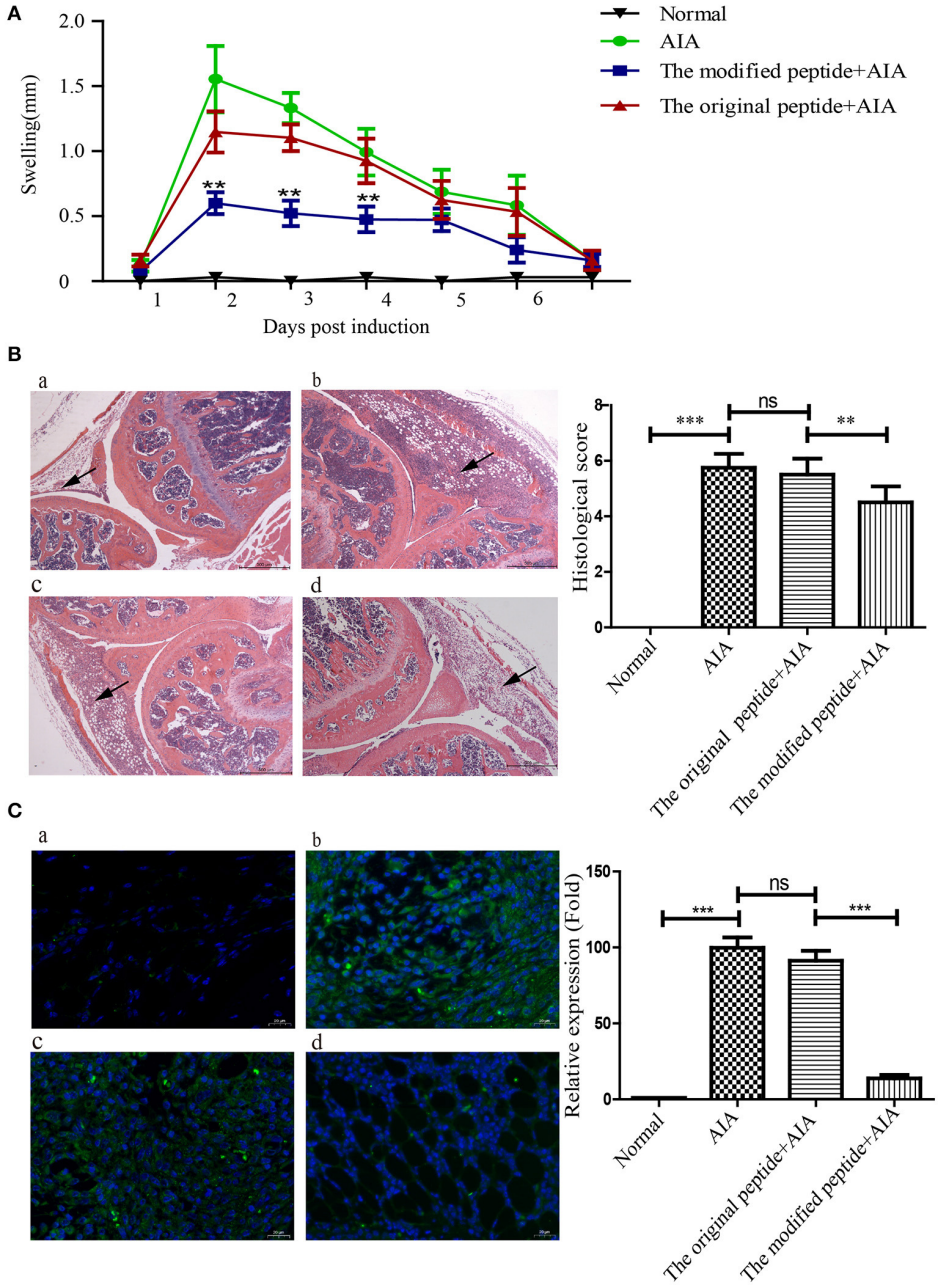
Modified peptide reduced joint inflammation and membrane attack complex (MAC) deposition in mice with antigen-induced arthritis (AIA). The right knee joint of each mouse was intraarticularly injected with 10 μl PBS as a control, while the left knee joint was injected with either 10 μl PBS only (normal group), 25 μg mBSA in 10 μl PBS (AIA-only group), 40 μg original peptide with 25 μg mBSA in 10 μl PBS (original peptide+AIA group), or 40 μg modified peptide with 25 μg mBSA in 10 μl PBS (modified peptide+AIA group). **(A)** Swelling of the left knee joint relative to the right knee joint was measured daily. Data are presented as mean ± SD. *n* = 6, **p < 0.01. **(B)** Representative histologic sections of murine knee joints stained with hematoxylin and eosin. a: normal group, b: AIA-only group, c: original peptide+AIA group, d: modified peptide+AIA group. *n* = 6. Magnification ×10. Arrowheads indicate the swollen synovial tissue. **(C)** Comparisons of C9 deposition in the knee joint sections among the four groups based on immunofluorescence assays. a: normal group, b: AIA-only group, c: original peptide+AIA group, d: modified peptide+AIA group. *n* = 6 ***p < 0.001, ns, not significant (*p* > 0.05).

To evaluate the potential of the modified peptide as a therapeutic agent, its effects on the physiologic course of AIA in mice were assessed by measuring the diameter of swollen joints and HE staining. As shown in Figures 5A,B, the HE stains of the AIA-only mice showed obviously joint swelling and inflammatory cell infiltration compared with the normal joint, indicating the successful establishment of AIA. The modified peptide+AIA mice showed significant reductions in joint swelling on days 2, 3, and 4 after disease induction compared to the AIA-only mice, while the original peptide+AIA mice showed a similar degree of swelling to the AIA-only mice at all three time points (Figure 5A). As shown in Figure 5B, the histologic outcomes concurred with the changes in the joint diameter. The synovial tissues of AIA-only and original peptide+AIA mice exhibited severe edema, hyperplasia, and inflammatory cell infiltration compared to normal joints. However, the modified peptide+AIA mice showed milder synovial hyperplasia and inflammatory cell infiltration compared to the AIA-only or original peptide+AIA mice. MAC deposition in the synovial connective tissue was clearly detected in the AIA-only mice and original peptide+AIA mice, but it was significantly weaker in the modified peptide+AIA mice, while the normal joints had no MAC deposition (Figure 5C).

## Discussion

RA is an autoimmune disease that has been confirmed to involve inappropriate complement activation in the joint cavity, making the delivery of anti-complement drugs into the joint cavity an ideal therapeutic strategy to relieve RA progression (Dijkstra et al., 2019). In animal models, intraarticular injection of complement inhibitors (such as soluble complement receptor 1 [sCR1], CD59 and anti-C5 neutralizing recombinant mini-antibody) prior to disease onset or at the time of disease flares has been shown to alleviate joint swelling and the development of AIA by inhibiting the activation of complement system at different stages (Harris et al., 2002; Williams et al., 2004; Durigutto et al., 2013). In our previous study, we identified a 14-amino acid peptide derived from *Ts*-pmy that had a similar bio-function to the original protein, as it inhibited the assembly of MAC, which is an important activated product of complement (Zhao et al., 2014). Herein, the *Ts*-pmy-derived peptide was modified with a cell membrane-targeting signal to extend its retention time so as to enhance its ability to inhibit complement activation in the final stage. The *in vivo* experiments further confirmed that the modified peptide exhibited therapeutic effectiveness in AIA in mice by preventing MAC assembly in the synovial tissue of joint cavities. As a unique kind of pharmaceutical compound, peptides can serve as small therapeutic reagents for treating various diseases. However, natural peptides tend to be rapidly cleared and have a short half-life and sometimes have low solubility (Kaspar and Reichert, 2013; Di, 2015; Lau and Dunn, 2018). Many strategies have been used to prolong their half-lives, such as N- or/and C-terminal modifications and replacement of L-amino acids (Di, 2015). A study reported that a unique membrane-targeting signal that is widely found in proteins (including HIV-1 Gag and myristoylated alanine-rich C-kinase substrate [MARCKS]) can non-specifically bind to cell membranes and help the proteins perform various functions on the outer cell membrane or endoplasmic reticulum (Murray et al., 1997). In this study, the unique membrane-targeting signal was linked to the peptide derived from *Ts*-pmy. MST assays revealed that the modified peptide had a binding affinity that was similar to that of the original peptide, and C9 polymerization and hemolytic assays further confirmed that the modified peptide could also inhibit MAC assembly (Figure 1). The results showed that the membrane-targeting signal did not interfere with the binding ability of the peptide to human C9, and its ability to inhibit complement activation was also retained. In addition, we showed that the original and modified peptides not only bind to human C9 but also to mouse C9, and they can inhibit the NMS-induced lysis of rabbit erythrocytes, suggesting that these peptides can cross the species barrier to inhibit MAC assembly in mice (Figure 2).

When assessing the membrane-targeting properties, we found that the modified peptide could non-specifically bind to the surface of mouse ATDC5 cells, as confirmed by immunofluorescence staining and flow cytometry (Figure 3). Furthermore, the *in vivo* experiments revealed that the articular cavities injected with the original peptide showed no staining, while those injected with the modified peptide showed intense staining along the synovial lining up to 48 h after injection (Figure 4), which is even longer than the report of a membrane-targeted soluble recombinant form of rat CD59 that remained on the synovial lining and subsynovial connective tissue for 24 h (Williams et al., 2004). The extended residence time of the modified peptide support its potential as a treatment for RA, as longer residence time in the joint cavity means improved inhibition of MAC formation.

Establishing a suitable disease model is key for assessing the effectiveness of drug treatment. Murine AIA, a well-recognized acute complement-dependent arthritis, imitates the pathology of RA (van den Berg et al., 2007). As complement activation is involved in the process of AIA, this model has been utilized in many studies to assess the therapeutic efficacy of complement inhibitors that might affect various stages of complement activation. In animal models, intraarticular injection of complement inhibitors (including sCR1, CD59, and anti-C5 neutralizing recombinant mini-antibody) prior to disease onset or at the time of disease flares has been shown to alleviate joint swelling and the development of AIA (Goodfellow et al., 2000; Williams et al., 2004; Durigutto et al., 2013). Similar to previous studies of the complement inhibitors' therapeutic effects on AIA, this study showed that inhibition of complement activation by intraarticular injection of the modified peptide at the time of AIA induction significantly decreased joint swelling, histopathological scores, and MAC deposition (as indicated by the decreased immunofluorescence) (Figure 5). The less therapeutic effect with original *Ts*-Pmy-derived peptide compared to the modified peptide shown in this study indicated that the membrane-targeting binding is crucial for the therapeutic efficacy of AIA.

As the terminal product of complement activation, MAC causes more than just osmotic lysis of target cells (Cole and Morgan, 2003). In the past few years, studies of the effects of MAC on nucleated cells, called sub-lytic lysis effects, have been revealed, including stimulation of inflammatory cytokines, chemokines, eicosanoids and adhesion molecules, proliferation, and apoptosis (Rus et al., 2001; Morgan, 2016). Analysis of human rheumatoid synovial cells attacked by MAC *in vitro* showed that the cells exhibited increased interleukin (IL)-6 secretion and prostaglandin E_2_ (PGE2) production (Morgan et al., 1988; Daniels et al., 1990a,b). In addition, using a rabbit AIA model, there was a significant decrease in synovial fluid neutrophils and IL-8 in C6-deficient rabbits compared to C6-sufficient rabbits, indicating the important roles of MAC in the recruitment and/or retention of inflammatory cells to inflamed joints (Tramontini et al., 2002). Herein, we simultaneously observed the decreased MAC deposition and joint inflammation in the modified peptide+AIA group compared to the original peptide+AIA and AIA+only groups (Figures 5B,C). Considering the relationship between MAC and the occurrence and development of inflammation, it is reasonable to believe that injection of the modified peptide inhibits MAC assembly and thereby delays the progress of inflammation and thus decreases the severity of AIA in the animal model. However, given the complexity and the large number of complement components, the original or modified peptide may also react with other complement components besides C9, inhibiting the activation of complement. The other potential targets of the peptides among the complement components need further investigation. Intra-articular injection with high concentrations of therapeutics is used in the treatment of osteoarthritis (OA) or RA patients (Rai and Pham, 2018). A recent study focus on the intra-articular drug delivery systems, which may remain in OA joints for a long time and sustainedly release drugs (Yang et al., 2014). However, the direct administration of therapeutics into joint cavity is limited in clinical application. The novel formulation and administration of *Ts*-Pmy-derived peptide in the treatment of RA should be further investigated.

Taken together, our results demonstrated that the membrane-targeting signal increases the retention time of the peptide at the injection site, so the modification may improve the peptide's therapeutic effects. The *Ts*-pmy-derived peptide modified with the membrane-targeting signal is a reasonable candidate therapeutic agent for MAC-related diseases such as RA.

## Data Availability Statement

The original contributions presented in the study are included in the article/supplementary materials, further inquiries can be directed to the corresponding author/s.

## Ethics Statement

The studies involving human participants were reviewed and approved by Institutional Review Board (IRB) of Capital Medical University (approval number: 2016SY01). The patients/participants provided their written informed consent to participate in this study. The animal study was reviewed and approved by Capital Medical University Animal Care and Use Committee (approval number: AEEI-2016-119).

## Author Contributions

XZ conceived and designed the experiments. YiC, SS, and JH performed the experiments. YiC, YuC, and YG analyzed the data. XZ, YiC, and YuC wrote the paper. All authors reviewed the manuscript. All authors contributed to the article and approved the submitted version.

## Conflict of Interest

The authors declare that the research was conducted in the absence of any commercial or financial relationships that could be construed as a potential conflict of interest.

## Supplementary Material

Supplementary Figure 1 Representative histologic sections of murine knee joints stained with hematoxylin and eosin. **(A)** The left knee of mouse injected with mBSA in AIA only group; **(B)** The right knee of mouse injected with PBS in AIA only group. Magnification ×10.
